# Identification of Crucial Amino Acids in Mouse Aldehyde Oxidase 3 That Determine Substrate Specificity

**DOI:** 10.1371/journal.pone.0082285

**Published:** 2013-12-16

**Authors:** Martin Mahro, Natércia F. Brás, Nuno M. F. S. A. Cerqueira, Christian Teutloff, Catarina Coelho, Maria João Romão, Silke Leimkühler

**Affiliations:** 1 Department of Molecular Enzymology, Institute of Biochemistry and Biology, University of Potsdam, Potsdam, Germany; 2 REQUIMTE, Departamento de Quimica, Faculdade de Ciencias, Universidade do Porto, Porto, Portugal; 3 Institute for Experimentalphysics, Free University of Berlin, Berlin, Germany; 4 REQUIMTE, Departamento de Química, Faculdade de Ciências e Tecnologia, Universidade Nova de Lisboa, Caparica, Portugal; National Institute for Medical Research, Medical Research Council, London, United Kingdom

## Abstract

In order to elucidate factors that determine substrate specificity and activity of mammalian molybdo-flavoproteins we performed site directed mutagenesis of mouse aldehyde oxidase 3 (mAOX3). The sequence alignment of different aldehyde oxidase (AOX) isoforms identified variations in the active site of mAOX3 in comparison to other AOX proteins and xanthine oxidoreductases (XOR). Based on the structural alignment of mAOX3 and bovine XOR, differences in amino acid residues involved in substrate binding in XORs in comparison to AOXs were identified. We exchanged several residues in the active site to the ones found in other AOX homologues in mouse or to residues present in bovine XOR in order to examine their influence on substrate selectivity and catalytic activity. Additionally we analyzed the influence of the [2Fe-2S] domains of mAOX3 on its kinetic properties and cofactor saturation. We applied UV-VIS and EPR monitored redox-titrations to determine the redox potentials of wild type mAOX3 and mAOX3 variants containing the iron-sulfur centers of mAOX1. In addition, a combination of molecular docking and molecular dynamic simulations (MD) was used to investigate factors that modulate the substrate specificity and activity of wild type and AOX variants. The successful conversion of an AOX enzyme to an XOR enzyme was achieved exchanging eight residues in the active site of mAOX3. It was observed that the absence of the K889H exchange substantially decreased the activity of the enzyme towards all substrates analyzed, revealing that this residue has an important role in catalysis.

## Introduction

Aldehyde oxidases (AOX, EC 1.2.3.1) are complex molybdo-flavoenzymes (MFEs) containing two distinct [2Fe-2S] centers, FAD and the molybdenum cofactor (Moco) as catalytically acting units. The enzymes are homodimers in eukaryotes and belong to the family of xanthine oxidases (XO) of molybdoenzymes. Despite their high sequence identity of about 50%, AOXs and xanthine oxidoreductases (XOR) use different sets of substrates. Both enzymes display a broad substrate specificity acting on activated carbon atoms in -C = N- and -C(-H) = O bonds [1], [2]. While XORs prefer low substituted purines as substrates, AOXs prefer higher substituted aromatic compounds. MFEs in general are characterized by a homodimeric butterfly-shaped structure of about 300 kDa in size. Three subdomains of each subunit containing three different cofactors characterize the structure of mammalian MFEs, a 20 kDa N-terminal [2Fe-2S] domain, a 40 kDa central FAD-containing domain and an 85 kDa C-terminal molybdenum cofactor (Moco) domain [3], [4]. The Moco of eukaryotic AOX and XOR is further modified by an equatorial sulfur ligand coordinating the molybdenum atom, which is essential for their catalytic function [5], [6]. The oxidation of substrates starts with a base-catalyzed nucleophilic addition of Mo-OH to the reducing substrate and concerted hydride transfer from the reducing substrate to the Mo = S moiety. The molybdenum is reduced from +VI to +IV in this reaction. The covalent intermediate is hydrolyzed by water releasing the oxidized product [7]. The electrons are transferred via the two [2Fe-2S] centers (FeSI nearest to the Moco and FeSII nearest to the FAD [8]) to the FAD cofactor. In AOX enzymes molecular oxygen is used as terminal electron acceptor, producing mainly H_2_O_2_. In XORs, the oxidase form (XO) uses O_2_ as electron acceptor, while the dehydrogenase form (XDH) prefers NAD^+^ as electron acceptor [9]. The inability of the oxidase form to reduce NAD^+^ has been assigned to structural changes upon oxidation or proteolytic cleavage that block the NAD^+^ binding site by sterical hindrance [10]. Alongside, the oxidase form shows a ∼170 mV more positive reduction potential of the FADH°/FADH_2_ couple in comparison to the dehydrogenase form, which preferentially yields the two electron reduced form of FAD being able to react with molecular oxygen [11]. So far, rabbit liver AOX is the only eukaryotic AOX for which redox potentials were determined [12]. The order of redox potentials of the FAD/FADH° couple and the FADH°/FADH_2_ couple of rabbit liver AOX was shown to be comparable to the one identified in XOs [9].

While XORs have a defined role in the catabolism of purines, the physiological role of AOXs is not known and only at the beginning to be understood [3]. Recent results imply a role in lipid-homeostasis and UV-light response [13]–[15] as well as pheromone reception [16]. While XORs are encoded by a single species-specific gene, for AOX enzymes different isoforms were identified in different species. Rodents and marsupials contain the largest number of *Aox* functional genes: *Aox1*, *Aox3*, *Aox4*, and *Aox3l1* [17], [18]. These genes arose from a series of gene duplication events from a common ancestor and are clustered on a short region of mouse chromosome 1 and rat chromosome 9. All of the products of the mammalian *Aox* genes have high amino acid sequence similarity and are expressed in a tissue-specific manner in different organisms [3], [19]. It is believed that the various AOX isoforms recognize distinct substrates and carry out different physiological tasks. The tissue distribution of mouse AOX3 (mAOX3) is superimposable to that of mAOX1, and the two enzymes are synthesized predominantly in liver, lung, and testis [20]. The expression of mAOX4 is limited to the Harderian gland, esophagus and skin, whereas mAOX3L1 expression is restricted to the nasal mucosa [21]. mAOX4 was shown to metabolize retinaldehyde into retinoic acid and plays a role in skin homeostasis [13]. Due to low-abundance and overlapping tissue-distribution purification of one AOX-isoform is difficult without any cross-contaminations of other AOX-isoforms. Therefore, little is known about substrate specificities and kinetic properties of the different AOX isoforms. Independently from the physiological role of AOXs, inter-species and inter-individual differences in AOX promoted drug metabolism has evolved to a challenging issue in drug development [22]. Understanding the mechanisms determining the different substrate specificities and the factors determining the reactivity of AOX is one of the main goals in this field. Recent recombinant expression systems in hand enabled to meet the increasing interest in understanding the molecular bases of AOXs substrate specificity.

In this study we use the existing recombinant expression system for mAOX3 in *Escherichia coli* to study the basis of catalytic activity and substrate selectivity of mAOX3 in comparison to other AOX homologues from mouse and bovine XOR (bXOR). We exchanged amino acids in the first and second coordination sphere of the mAOX3 active site to amino acids found in XOR and other AOX isoforms. The variant mAOX3-F776K/A807E/D878L/L881S/Y885R/P1015T/Y1019L showed drastically decreased activity towards all substrates tested. Additional introduction of K889H substitution rescued AOX activity and resulted in a XOR-like activity with hypoxanthine as substrate. Additionally, we introduced the iron-sulfur center domains of mAOX1 in mAOX3. Changes in activity as well as iron-sulfur center I (FeSI) formal potentials were observed.

## Results and Discussion

### Site-directed mutagenesis of mAOX3

**Figure 1:**
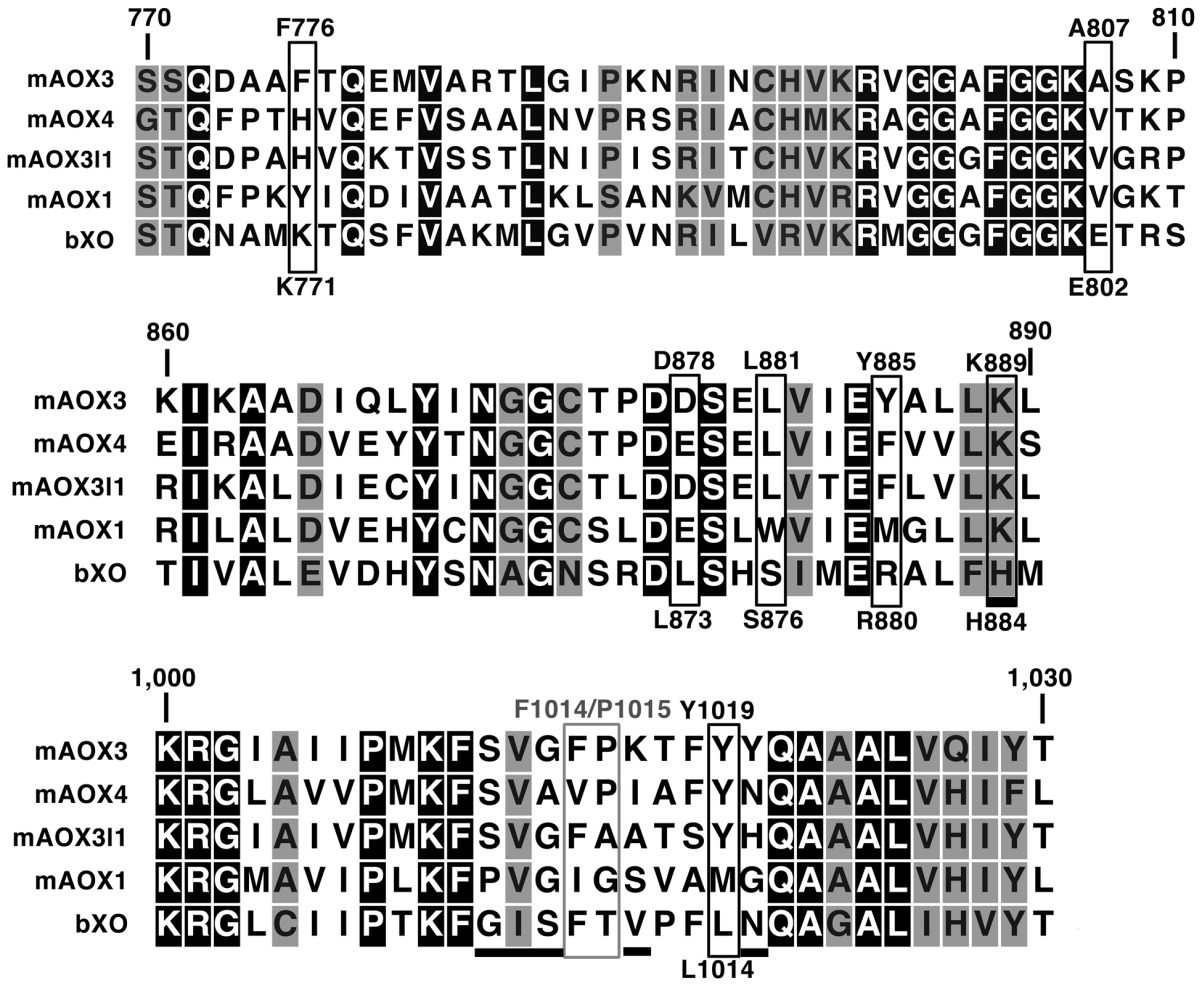
Amino acid sequence alignment of selected parts of mAOX homologues and bovine XOR. Shown is an amino acid sequence alignment of the region of amino acids 770-1030 of mAOX3 with the other AOX-isoform from mouse and bovine XOR (bXOR). Identical amino acid residues are shaded in black, homologous amino acid residues are shaded in grey. For clarity, residues exchanged in mAOX3-“active site1”-K889H variant are boxed in black. A grey box marks the position of mAOX3-F1014 and mAOX3-P1015 variants. The six residues exchanged in the mAOX3-“gating loop” variants are underlined. The alignment was created with CLC Sequence viewer Ver. 6.8.2.

**Figure 2:**
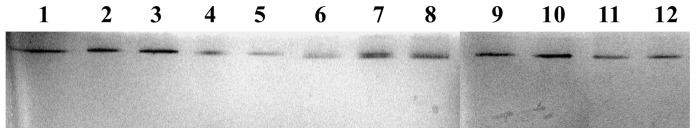
8% native-PAGE of mAOX3 wild type and variants. Lanes 1–12 contain 3–5 µg of purified protein, stained with Coomassie-brilliant blue. 1, mAOX3 WT; 2, mAOX3-“active site1”; 3, mAOX3-“active site1”-K889H; 4, mAOX3-“active site1 + gating loop”; 5, mAOX3-“gating loop”; 6, mAOX3-FeSI-mAOX1; 7, mAOX3-FeSII-mAOX1; 8, mAOX3-FeSII-FeSI-mAOX1; 9, mAOX3-P1015A; 10, mAOX3-P1015G; 11, mAOX3-F1013I; 12, mAOX3-F1014V.

In order to determine the role of different amino acids in the active sites of AOXs, the variants mAOX3-F1014I, mAOX3-F1014V, mAOX3-P1015A and mAOX3-P1015G were generated. Additionally, we performed “whole domain” exchanges to study differences in the intramolecular electron transfer. We exchanged the [2Fe-2S]-containing domains of mAOX3 to the ones present on mAOX1 to study their influence on electron transfer and catalytic activity of mAOX3. The variant mAOX3-FeSII-mAOX1 exhibits the amino acids M1 – P76 from mAOX1 instead of M1 – P80 of mAOX3. In mAOX3-FeSI-mAOX1, A87 – Q214 of mAOX3 is exchanged by T83 – Q213 of mAOX1 and mAOX3-FeSII-FeSI-mAOX1 possesses M1 – Q213 from mAOX1 replacing M1 – Q214 from mAOX3. In order to study amino acids, which determine the substrate specificity of XOR enzymes in comparison to AOX enzymes, we performed numerous exchanges of amino acids in the first and second coordination sphere of the active site of mAOX1 to the ones present in bXOR. The variant named mAOX3-“active site1” contains the seven amino acid exchanges F776K/A807E/D878L/L881S/Y885R/P1015T/Y1019L in mAOX3, which represent highly conserved amino acids in XOR enzymes (Fig. 1). The mAOX3-“active site1”-K889H variant contains in addition the K889H exchange, since the lysine was shown to be involved in substrate binding in AOX enzymes [23]. All variants named “gating loop” contain the amino acid exchanges K889H/S1011G/V1012I/G1013S/K1016V/Y1020N, which are in the vicinity of the active site. These amino acids are part of a hydrogen-bond network in the second coordination sphere of XORs, which is mainly build by R880 (Y885 in mAOX3), G1006-S1008 (S1011-G1013 in mAOX3) and N1015 (Y1020 in mAOX3). All 11 mAOX3 variants were expressed and purified from 28 – 112 liter cultures as described previously for mAOX3 wild type [24]. Figure 2 shows wild type mAOX3 and the purified variants separated on an 8% native polyacrylamide gel. All variants were purified as dimers with a comparable yield of 0.5 – 1.2 mg/L of culture and a purity of approx. 95%. As shown in Table 1, all variants showed a comparable molybdopterin (MPT) saturation of 62±24% – 139±37% relative to mAOX3 wild type. The iron content was found to be similar with a saturation ranging from 49±15% to 75±24%. The ratio of absorbance at 444 nm and 550 nm was around 3.2 for all variants, showing an equal ratio of FAD to the 2x[2Fe-2S] clusters. Molybdenum saturation ranged from 45±8% to 101±16%. The molybdenum to MPT ratio in all variants was found to be comparable to the mAOX3 wild type, showing an unaltered amount of the demolybdo form of MPT present in the purified proteins. The ratio of absorbance at 280 nm and 444 nm in the UV-Vis spectra of all variants were identical in the range of 4.9 – 5.7, showing an almost 100% saturation of the variants with FAD [24].

**Table 1:** Determination of the cofactor composition of the mAOX3 variants.

	% Mo content^1^	% Fe content^1^	% MPT content^2^	A_280_/A_444_	A_444_/A_550_
**mAOX3-WT**	62±13	57±7	100±10	5.2	3.3
**mAOX3-F1014I**	70±9	65±17	62±24	5.2	3.1
**mAOX3-F1014L**	45±8	49±15	1006	5.2	3.2
**mAOX3-P1015A**	55±9	75±24	105±2	5.7	3.1
**mAOX3-P1015G**	60±4	67±1	116±2	5.4	3.2
**mAOX3-FeSI-mAOX1**	79±4	64±1	106±10	5.6	3.4
**mAOX3-FeSII-mAOX1**	96±12	73±18	139±37	5.6	3.1
**mAOX3-FeSII-FeSI-mAOX1**	101±16	69±10	128±14	5.7	3.3
**mAOX3-“active site1”**	46±1	52±0	96±3	5.1	3.5
**mAOX3-“active site1”-K889H**	63±3	63±0	87±11	4.9	3.3
**mAOX3-“active site1 + gating loop”**	85±6	58±3	73±22	5.4	3.2
**mAOX3-“gating loop”**	60±2	66±1	75±2	5.1	3.3

µM molybdenum/µM enzyme) and iron (µM 2 x [2Fe2S]/µM enzyme) contents were determined by ICP-OES (see Experimental procedures).^1^ Molybdenum (

%.^2^ Determined after conversion to the stable oxidized fluorescent product FormA. mAOX3-WT was set to 100

Relative molybdopterin (MPT) saturation, molybdenum and iron saturation and ratio of absorbance at 280 nm, 444 nm and 550 nm of mAOX3 variants in comparison to reported values for the wild type enzyme.

### Analysis of the role of residues F1014 and P1015 in mAOX3

The crystal structure of mAOX3 suggested that A807, Y885, K889, F919 and F1014 are directly involved in substrate-binding in mAOX3 [23]. An amino acid sequence alignment of mAOX3 with mAOX1, mAOX4, mAOX3I1 and bovine XOR showed differences in conservation of residues in the active site among the enzymes (Figure 1). The residues A807 and Y885 present in mAOX3 are highly conserved in AOX1 enzymes and are replaced by valine and a methionine in other isoforms, respectively (Figure1). F919 is highly conserved between all vertebrate AOX and XOR enzymes, while F1014 is only conserved in XOR enzymes and varies among AOX homologues. To study the role of F1014 of mAOX3, we exchanged it to an isoleucine (present in mAOX1) and a valine (present in mAOX4) [18]. The adjacent amino acid P1015 is also not conserved. To study its role, we exchanged mAOX3-P1015 to a glycine (present in mAOX1) and to an alanine (present in mAOX3l1). The catalytic constants of mAOX3 wild type, mAOX3-F1014I, mAOX3-F1014V, mAOX3-P1015A and mAOX3-P1015G are shown in Table 2. All values were corrected for a molybdenum saturation of 100% of each variant for a better comparability. In comparison to mAOX3 wild type, the variant mAOX3-F1014I showed a 7.4–33 fold decreased catalytic efficiency (k_cat_/K_M_) with benzaldehyde, phthalazine and phenanthridine. Both, the substrate affinity and rate of conversion were affected by the F1014I exchange. mAOX3-F1014I was determined to be inactive with allopurinol and N1-methylnicotinamide as substrates. Replacement of F1014 by valine resulted in a 2.1–11 fold decrease in catalytic efficiency with all substrates. However, the K_M_ values of the mAOX3-F1014V variant were higher in comparison to wild type mAOX3. In contrast, the k_cat_ with benzaldehyde and phthalazine was increased, while the k_cat_ with N1-methylnicotinamide and phenanthridine remained unchanged. Allopurinol was oxidized with a 4.6 - fold lower k_cat_ by mAOX3-F1014V in comparison to mAOX3 wild type. The catalytic constants were also altered by variation of P1015 to alanine or glycine. The catalytic efficiency of mAOX3-P1015A and mAOX3-P1015G with benzaldehyde and phthalazine was comparable to mAOX3 wild type. N1-methylnicotinamide was converted with a higher catalytic efficiency by variants mAOX3-P1015A and mAOX3-P1015G in comparison to mAOX3 wild type. The catalytic efficiency (k_cat_/K_M_) with phenanthridine was higher for the variants mAOX3-P1015A and mAOX3-P1015G in comparison to wild type mAOX3. The increase in catalytic efficiency with allopurinol was comparable for both mAOX3-P1015 variants.

**Table 2:** Steady-state kinetic parameters of mAOX3 wildtype and mAOX3 variants containing single amino acid exchanges in the active site.

substrate	kinetic parameters^3^	mAOX3-WT^4^	mAOX3-F1014I	mAOX3-F1014V	mAOX3-P1015A	mAOX3-P1015G
**benzaldehyde** 1	*kcat [min^−1^]*	68±15	19±3	128±12	76±14	93±7
	*KM [µM]*	2.5±0.2	22.7±2.7	21.5±3.2	2.2±0.1	3.3±0.2
	*kcat/KM [min^−1^ µM^−1^]*	27.0±6.4	0.8±0.2	5.9±1.0	34.7±6.4	28±2.7
**phthalazine** 1	*kcat [min^−1^]*	66±15	58±9	278±14	179±38	347±64
	*KM [µM]*	1.4±0.2	9.0±0.5	12.5±0.6	2.6±0.3	10.3±2
	*kcat/KM [min^−1^ µM^−1^]*	47.3±12.7	6.4±1.0	22.3±1.5	68.9±16.6	33.7±9
**phenanthridine** 2	*kcat [min^−1^]*	85±18	11±3	85±11	581±153	530±49
	*KM [µM]*	32±1	73±18	385±70	99±15	47±2
	*kcat/KM [min^−1^ µM^−1^]*	2.63±0.58	0.16±0.05	0.22±0.05	5.88±1.8	11.37±1.19
**N1-methylnicotineamide** 1	*kcat [min^−1^]*	24±5	n.d.	23±1	65±11	115±8
	*KM [µM]*	128±5.8	n.d.	482.3±28	87±3.4	88.7±2.3
	*kcat/KM [min^−1^ µM^−1^]*	0.185±0.4	-	0.047±0.004	0.743±0.132	1.297±1.101
**allopurinol** 1	*kcat [min^−1^]*	73±16	n.d.	16±2	46±9	37±4
	*KM [µM]*	1595±73	n.d.	3860±567	527±63	426±56
	*kcat/KM [min^−1^ µM^−1^]*	0.0457±0.0103	-	0.0041±0.0008	0.0871±0.0203	0.0864±0.0145

µM DCPIP was used as terminal electron receptor.^1^ 100

^2^ Molecular oxygen in air saturated buffer was used as terminal electron acceptor.

°C; k_cat_ was corrected for molybdenum content^3^ Determined in 50 mM Tris (HCl) pH 8.00 at 37

[23] and corrected for molybdenum content.^4^ Values from

n.d.: non detectable

In summary, exchange of F1014 to valine or isoleucine resulted in a decrease in substrate affinity and catalytic efficiency with all substrates tested. Thus, valine and isoleucine might interfere with substrate binding due to steric interferences. The increase in K_M_ is consistent with the higher K_M_ values determined for mAOX1 containing an isoleucine at this position with these substrates [25]. In contrast, the higher rate constants with benzaldehyde and phthalazine obtained for mAOX1 were not observed with the mAOX3-F1014I variant, however, exchange of mAOX3-P1015 by alanine or glycine resulted a higher k_cat_ with bulky and charged substrates. These changes might be based on two effects in the mAOX3-P1015 variants. The smaller side chain of alanine and glycine increases the flexibility of the peptide backbone and the ability of Y885 to flip away from the substrate, as suggested previously [23]. This might result in a better access of the substrate to K889, thereby resulting in tighter binding and/or better stabilization of the transition state. However, there is an up to 30-fold difference in the catalytic efficiency with benzaldehyde between mAOX1 (k_cat_/K_m_  = 3.34±0.56 min^−1^µM^−1^), hAOX1 (k_cat_/K_m_  = 0.91±0.17 min^−1^µM^−1^) and mAOX3 (k_cat_/K_m_  = 27.03±6.37 min^−1^µM^−1^) [23], [25], [26]. While the mAOX3-F1014I variant resembled the variation of catalytic efficiency between mAOX1 and mAOX3, the up to 20-fold difference in the rate constant between mAOX1, mAOX3 and hAOX1 was not observed by a single variation of F1014 or P1015. Thus, more factors might contribute to the difference in the catalytic constants of the AOX homologues.

### The role of FeSI and FeSII on electron transfer and activity of mAOX3

To analyze the effect of the two [2Fe2S] clusters for substrate conversion in AOX enzymes, we generated three variants by exchanging FeSI, FeSII and both FeSI/FeSII in mAOX3 by the domains of mAOX1. The kinetic parameters (Table 3) of the iron-sulfur domain variants were determined using molecular oxygen (with the substrate phenanthridine) or DCPIP as terminal electron acceptors in order to exclude mediator-based side effects. As shown in Table 3, the exchanges of the iron-sulfur center domains in mAOX3 to the ones present in mAOX1 did result in a decrease in catalytic activity with all substrates tested. A decrease of reaction rate was obtained for both electron acceptors, DCPIP (accepting electrons from Moco [27], [28]) and O_2_ (reacting with the FAD site). The effect on catalytic activity was less pronounced for the FeSI variant, while the highest decrease in the catalytic activity was observed for the variant containing both iron-sulfur domains of mAOX1. Additionally, we determined the reduction potentials of the cofactors present in the protein (Table 4). The reduction potentials for the FAD/FADH_2_ redox pairs were found to be similar in all variants. These values are close to the FAD°/FADH_2_ reduction potential determined for rabbit AO (−212 mV) [12]. No detectable amount of FAD-semiquinone was observed during the titration process. Exchange of the iron-sulfur domains had only minor effects on the reduction potential of FeSII. By exchange of FeSI or FeSI/FeSII to the ones found in mAOX1, a lower reduction potential of FeSI was observed. The reduction potentials of the MoIV/V and MoV/VI redox couples in mAOX3 wild type were found to be similar for all three iron-sulfur domain mAOX3-variants (Table 4). A linear correlation according to the Marcus theory was only observed for the reduction potential difference between the MoV/VI redox pair and FeSI, with values for the electronic coupling matrix of H_AB_  = 5.76×10^−16^±2.14×10^−16^ and reorganization energy of λ  = 2.57×10^4^±6.84×10^3^ J/mol. A previous report by Itoh et al. [29] examined the effect of Moco domain swop between monkey and rat AOX on the conversion of (S)-RS-8359. The authors observed that the substrate affinity and susceptibility to substrate inhibition of the chimeric variants was equal to the enzyme from which the Moco domain originated. The rate constant of the chimeric variants on the other hand was equal to the enzyme from which the iron-sulfur/FAD part originated. It was concluded that the rate of conversion is controlled by the iron-sulfur/FAD part, whereas the Moco domain defines substrate affinity and susceptibility to substrate inhibition. Our results show that substrate affinities of all iron-sulfur domain variants were unchanged. Exchange of the FeSII domain influenced the reduction potential of FeSI, but not the reduction potentials of Moco or FAD. Thus, in consistence with the results of the domain swapping of the rat enzyme, the rate of substrate conversion is not determined by the redox potential of the molybdenum atom. It is rather determined by structural effects induced by the domain swapping of the FeS domains influencing the electron transfer reaction by a change of the redox potential of the FeS clusters.

**Table 3:** Steady-state kinetic parameters of mAOX3 wild type and mAOX3 variants containing the FeS-domains of mAOX1.

substrate	kinetic parameters^3^	mAOX3-WT^4^	mAOX3-FeSI-mAOX1	mAOX3-FeSII-mAOX1	mAOX3-FeSII-FeSI-mAOX1
**benzaldehyde** 1	*k_cat_ [min^−1^]*	68±15	48±3	38±5	23±4
	*K_M_ [µM]*	2.5±0.2	3.6±0.3	1.6±0.1	2.2±0.1
	*k_cat_/K_M_ [min^−1^ µM^−1^]*	27±6.4	13.4±1.5	23.7±1.9	10.3±1.9
**phthalazine** 1	*k_cat_ [min^−1^]*	66±15	45±3	32±5	30±5
	*K_M_ [µM]*	1.4±0.2	1.2±0.1	1.4±0.2	1.0±0.1
	*k_cat_/K_M_ [min^−1^ µM^−1^]*	47.3±12.7	38.3±3.9	22.9±4.8	29.1±5.3
**phenanthridine** 2	*k_cat_ [min^−1^]*	85±18	43±3	26±4	25±4
	*K_M_ [µM]*	32±1	35±2	30±5	28±2
	*k_cat_/K_M_ [min^−1^ µM^−1^]*	2.63±0.58	1.23±0.1	0.88±0.21	0.87±0.17
**allopurinol** 1	*k_cat_ [min^−1^]*	73±16	25±2	20±4	10±2
	*K_M_ [µM]*	1595±73	1338±43	899±145	1026±108
	*k_cat_/K_M_ [min^−1^ µM^−1^]*	0.0457±0.0103	0.0185±0.0013	0.022±0.0055	0.01±0.0022

µM DCPIP was used as terminal electron receptor.^1^ 100

^2^ Molecular oxygen in air saturated buffer was used as terminal electron acceptor.

°C; k_cat_ was corrected for molybdenum content^3^ Determined in 50 mM Tris HCl pH 8.00 at 37

[23] and corrected for molybdenum content.^4^ Values from

**Table 4:** Reduction potentials of mAOX3 and selected variants determined by electron paramagnetic resonance spectroscopy and UV/Vis titrations.

	Mo IV/V	Mo V/VI	FeSI	FeSII	FAD/FADH_2_
mAOX3-WT	−468±11	−294±9	−206±10	−286±12	−185±11
mAOX3-FeSI-mAOX1	−44065	−234±33	−198±14	−230±21	−180±11
mAOX3-FeSII-mAOX1	−441±38	−290±43	−273±22	−260±23	−201±11
mAOX3-FeSII-FeSI-mAOX1	−422±28	−31329	−305±13	−261±22	−174±12

Proteins were titrated anaerobically with sodium dithionite at room temperature in the presence of mediators as enlisted in experimental procedures. Signal intensities were determined by weighted fit of simulated spectra to experimental data. Weight parameters plotted against electrode potential were fitted to the Nernst-equation as described in experimental procedures. Reduction potentials of FeSI are lower in mAOX3 variants exhibiting iron sulfur domains from mAOX1.

### Change of substrate specificity of mAOX3

**Figure 3:**
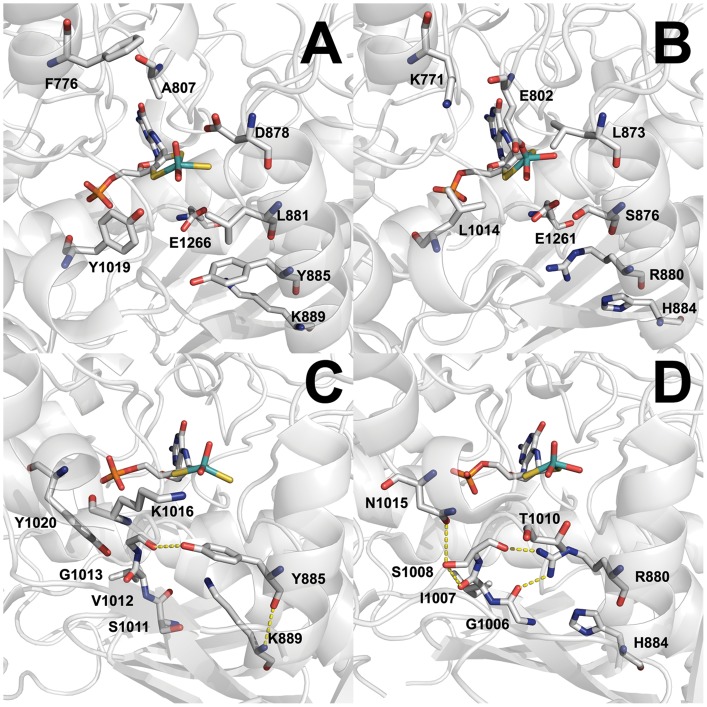
Active site structure of mAOX3. A, Stick representation of E1266 and residues exchanged in mAOX3-“active site1”-K889H in the crystal structure of mAOX3 WT (pdb:3ZYV). B, Stick representation of residues of desulfurated bXO (pdb:3EUB) corresponding to the amino acids in Panel A. C, Stick representation of residues in mAOX3 corresponding to the residues shown in Panel D. Y885 builds hydrogen-bonds to the backbone of G1013 and K889, indicated by yellow dotted lines D, Stick representation of residues involved in a hydrogen network at the entrance to the active site of bXOR. The hydrogen-bonding network is established by R880, G1006, I1007, S1008 and N1015 in bXOR represented by yellow dotted lines. Through the interactions, the position and orientation of T1010 is altered in comparison to K1016 of mAOX3 in panel C. Figures were created using MacPymol [56]Ver. 0.99rc6.

The main amino acids involved in substrate binding in bXOR were shown to be E802 (A807 in mAOX3) and R880 (Y885 in mAOX3) [10], [30]. The highly conserved residues F914 (F919 in mAOX3) an F1009 in bXOR (F1014 in mAOX3) were shown to provide additional van-der-Waals interactions to the substrate [23], [31]. In AOX enzymes a conserved lysine (K889 in mAOX3) was proposed to be involved in substrate binding, which is a histidine in XORs. As reported previously, the mAOX3-K889H exchange decreased the catalytic activity 2–4 - fold, with lower K_M_ values for most substrates, which suggested a better access of substrates to the active site due to the smaller side-chain [23]. We generated the variant mAOX3-“active site1” in which all residues in the first coordination sphere around the substrate except K889 were exchanged to their counterparts in XOR (F776K/A807E/D878L/L881S/Y885R/P1015T/Y1019L). The resulting variant mAOX3-“active site1” was devoid of activity towards most substrates tested. Only allopurinol was oxidized at a very low but significant rate of 14±1 min^−1^ (Table 5). However, additional introduction of the exchange of K889 to histidine in this variant recovered the activity with benzaldehyde, and phthalazine and, additionally, was able to convert hypoxanthine with a k_cat_ of 25±1 min^−1^. Benzaldehyde and phthalazine were oxidized with higher rate constants in comparison to mAOX3 wild type, however, the K_M_ values were largely increased. Thus, the additional introduction of K889H converted mAOX3 to a “XOR-type” enzyme (Figure 3). In order to test the additional effects of amino acids building a hydrogen-bond network in the second coordination sphere, we tested the “gating loop” variant (K889H/S1011G/V1012I/G1013S/K1016V/Y1020N) on substrate binding, in addition to variant mAOX3-“active site1 + gating loop”, which contains amino acid exchanges in the first and second coordination sphere (F776K/A807E/D878L/L881S/Y885R/K889H/S1011G/V1012I/G1013S/P1015T/K1016V/Y1019L/Y1020N). The affinity of mAOX3-“active site1 + gating loop” towards allopurinol, benzaldehyde and phthalazine was increased 3.7–22 fold in comparison to the variant mAOX3-“active site1”-K889H. On the other hand, the catalytic activity decreased 2–12 fold for these substrates. The catalytic efficiency of the mAOX3-“active site1 + gating loop” towards hypoxanthine was determined with values 5 times higher in comparison to mAOX3 wild type, but 2 times lower to that of the mAOX3-“active site1”-K889H variant. For comparison, a mAOX3 variant containing only the “gating loop” showed no detectable activity towards allopurinol, hypoxanthine and xanthine and only residual activity (1.3% in comparison to mAOX3 wild type) with benzaldehyde and phthalazine as substrates, showing that the additional amino acid exchanges are required to obtain a considerable activity. Thus, introduction of the “gating loop” exchange increased the substrate affinity in comparison to mAOX3-“active site1”-K889H, but resulted in a decrease in substrate conversion of the enzyme.

**Table 5:** Steady-state kinetic parameters for mAOX3 wild type and mAOX3 variants containing numerous amino acids present in the active site of bovine XOR.

substrate	kinetic parameters^1^	mAOX3-WT^2^	mAOX3-“active site1”	mAOX3-“active site1”-K889H	mAOX3-“active site1 + gating loop” 4	mAOX3-“gating loop”
**benzaldehyde**	*kcat [min^−1^]*	68±15	n.d.	234±15	53±5	0.9±0.2
	*KM [µM]*	2.5±0.2	n.d.	2122±88	571±36	4.5±3.4
	*kcat/KM [min^−1^ µM^−1^]*	27±6.4	-	0.110±0.008	0.092±0.011	0.199±0.151
**phthalazine**	*kcat [min^−1^]*	66±15	n.d.	524±33	42±7 (359±35)4	0.9±0.1
	*KM [µM]*	1.4±0.2	n.d.	733±52	33±18 (26806±2214)4	0.9±0.5
	*kcat/KM [min^−1^ µM^−1^]*	47.3±12.7	-	0.715±0.068	1.287±0.744 (0.013±0.002)4	0.997±0.559
**hypoxanthine**	*kcat [min^−1^]*	2.1±0.4	n.d.	25±1	5±0	n.d.
	*KM [µM]*	3685±854	n.d.	4085±227	1736±155	n.d.
	*kcat/KM [min^−1^ µM^−1^]*	0.00057±0.00018	-	0.00624±0.00048	0.00287±0.00035	-
**allopurinol**	*kcat [min^−1^]*	73±16	14±1	95±6	39±3	n.d.
	*KM [µM]*	1595±73	2933±380	133±10	620±16	n.d.
	*kcat/KM [min^−1^ µM^−1^]*	0.0457±0.0103	0.005±0.001	0.718±0.067	0.063±0.005	-

µM DCPIP in 50 mM Tris (HCl) pH 8.00 at 37°C; k_cat_ was corrected for molybdenum content^1^ Determined in the presence of 100

[23] and corrected for molybdenum content.^2^ Values from

n.d. none detectable.

^4^ phthalazine:DCPIP reaction was biphasic.

### Molecular Docking

**Figure 4:**
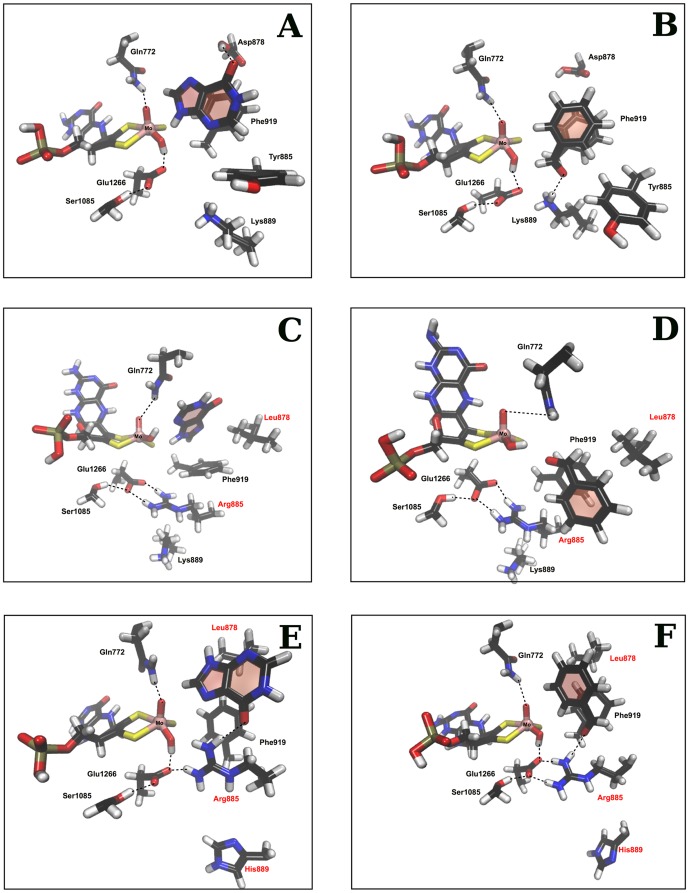
Molecular docking and molecular dynamic studies. A, Wild type enzyme with hypoxanthine. B, Wild type enzyme with benzaldehyde bound in the active site. C, mAOX3-“active site1” variant with hypoxanthine. D, mAOX3-“active site1” variant with benzaldehyde bound in the active site. E, mAOX3-“active site1”-K889H with hypoxanthine. F, mAOX3-“active site1”-K889H with benzaldehyde bound in the active site. Hydrogen bonds are marked with a dashed line and the altered residues are highlighted in red.

To interpret the results of the kinetic analyzes, we performed molecular docking analyzes. We have first docked hypoxanthine and benzaldehyde into the active sites of the wild type and mAOX3 variants by using the molecular docking software AutoDock (Fig. 4 A and B). The most favorable complexes were then regarded as the initial geometries for the following MD simulations. The computational results showed that in the mAOX3 wild type, the position of the molybdenum coordination is partially stabilized by two important hydrogen bonds that are established with Q772 and E1266. Q772 established one hydrogen bond with the axial oxygen that is bound to the molybdenum ion (on average 2.50 Å) and E1266 interacts very closely with the hydroxyl group of the metal complex through a short hydrogen bond (on average 2.00 Å). S1085 and K889 stabilize the anionic nature of E1266. S1085 interacts with E1266 by a short hydrogen bond (on average 2.50 Å) while the K889 forms an ionic bridge with it (on average 3.50 Å).

The computational results for the docking of hypoxanthine to mAOX3 wild type (Fig. 4A) showed a binding of the imidazole ring pointing towards the hydroxyl group of the molybdenum atom (2.04±0.20 Å). The position of hypoxanthine in the active site is stabilized by D878 trough a hydrogen bond involving the carbonyl group and by F919 via π-stacking interactions (∼3.35 Å). The modeled position of benzaldehyde in the active site is similar to the binding position of hypoxanthine (Fig. 4B). The aldehyde group resides close to the hydroxyl group of the Mo-OH group (3.46±0.18 Å) and is positioned correctly in order to be oxidized to the corresponding carboxylic acid. The conformation adopted by benzaldehyde is also guided by the π-stacking interactions with F919 (∼3.50 Å) and especially by K889, which establishes a hydrogen bond (3.13±0.60 Å).

The computational results for the mAOX3-“active site1” variant showed that the metal cluster is deviated from the position that it occupies in the wild type enzyme. This occurs due to the disruption of key hydrogen bonds between the metal complex and E1266 or Q772. This fact is supported by a movement E1266 that no longer interacts with the hydroxyl group of the metal complex. The new conformation adopted by E1266 is endorsed by the ionic bridge provided by the Y885R variation (2.50 Å), but also by the close proximity of K889. We believe that the proximity of R885 and K889 in the active site creates a strong positively charged space that attracts the negatively charged E1266. All of these factors together might result in an inactive enzyme.

The binding of hypoxanthine in the mAOX3-“active site1” variant is very different from the wild type enzyme (Figure 4C). The molecule has now flipped by 180° in the active site and the imidazole ring no longer interacts with the metal cluster. The D878L variation additionally precludes any interaction of the substrate with this part of the active site. In the wild type, the aspartate is responsible for making a hydrogen bond with the carbonyl group of hypoxanthine. The binding position of benzaldehyde in the mAOX3-“active site1” variant is also different from what is observed in the wild type enzyme (Figure 4D). However, in this case, the aldehyde group remained aligned with the hydroxyl group of the metal complex. The computational results suggest that the loss of activity might be related to the absence of the key interactions between the hydroxyl group and E1266.

The additional introduction of the K889H exchange to the mAOX3-“active site1” variant resulted in an activity towards benzaldehyde and hypoxanthine. The studies showed that the overall rearrangement of the residues of the active site in the mAOX3-“active site1”-K889H variant closely resembled that of the wild type enzyme. The metal complex continues to be firmly aligned in the active site with the hydroxyl group pointing towards the entrance of the active site tunnel provided by E1266 and Q772. The main differences between the mAOX3-“active site1”-K889H variant and the wild type enzymes are at the top and bottom regions of the active site where the substrate binds. The variation of D878 to a leucine turns the top region of the active site almost hydrophobic, similarly to what is found in the mAOX3-“active site1” variant. Consequently, any interaction between the substrate and this region is precluded. At the bottom of the active site, the variations of Y885 to an arginine and K889 to a histidine, did not lead to any significant rearrangement of the active site, except to the closer ionic interaction between E1266 and R885. Interestingly, in this case the interaction between E1266 and the hydroxyl group of the metallic center is not destroyed. Here, K889H removes the high concentration of positive charge in a specific region of the active site. The binding poses of hypoxanthine in the variant and wild type enzymes are very similar (Fig. 4E). The imidazole ring remained close to the hydroxyl group of the metallic center (3.76±039 Å) but the molecule has flipped 180°. Thus, the carbonyl group no longer interacts with the top part of the active site, and establishes a hydrogen bond with R885 (1.98±0.22 Å). This shows that the important role of D878 in the wild type enzyme was replaced by R885 in the variant enzyme. Interestingly, in the wild type enzyme K889 occupies a similar position that is occupied by R885 in the mAOX3-“active site1”-K889H and bXOR. However in that case, the interaction between hypoxanthine and D878 is preferred instead. The binding poses of the benzaldehyde in the wild type and the mAOX3-“active site1”-K889H variant are also very similar (Fig. 4F). The aldehyde group is in close contact to the hydroxyl group of the metal complex. The changes in the orientations of the substrate are not observed here, since benzaldehyde does not interact with D878. However, in the mAOX3-“active site1”-K889H variant the presence of R885 allows the carbonyl group of the aldehyde to establish a hydrogen bond with this residue. Thus, this variation might improve the substrate recognition and turn the active site more prone for this type of substrates. This explains the improved activity of mAOX3-“active site1”-K889H with benzaldehyde.

### Conclusions

AOX isoforms share a high amino acid sequence identity, however, their substrates specificities differ. Our results show that single variations of F1014 and P1015 in mAOX3 to residues found in mouse AOX homologues cannot explain the differences in the kinetic parameters to their full extent. Thus, more amino acid residues or synergistic effects determine the specificities and differences in the active site of these enzymes.

This work also shows that exchange of iron-sulfur domains influence the rate constant but not the substrate affinity of mAOX3. This is in accordance with Moco-domain swop experiments performed with monkey and rat AOX orthologs [29]. A correlation of k_cat_ and the difference in reduction potential between the Mo V/VI pair and FeSI was observed. However, our data do not allow favouring changes in the electron transfer or structural changes induced by the domain swop as a cause for the altered rate constants. Given the interaction at the interface of mAOX3 subdomains it seems impossible to alter the electron transfer without inducing structural changes.

Additionally, XOR and AOX enzymes act on different, yet overlapping sets of substrates, and XOR enzymes usually show higher rate constants towards their specific substrates. Here, we present for the first time the successful conversion of an AOX enzyme to an XOR enzyme that can convert hypoxanthine as substrate. This change in substrate specificity was obtained by exchange of several amino acids in the substrate binding site. The results show the importance of K889 in retaining the interaction between E1266 and the hydroxyl group of the molybdenum atom (see Table 6 for a more detailed analysis). Thus, the inactivity of the AOX3-“active site1” variant is caused mainly by the disruption of a key hydrogen bond between E1266 and the hydroxyl group of the molybdenum site, a reaction essential to start the base-catalyzed mechanism. By additional introduction of the K889H exchange, the hydrogen bond network remained established. Thus, the results show that the conversion of the AOX to a XOR enzyme observed for the mAOX3-“active site1”-K889H variant is only possible because the interaction between E1266 and the hydroxyl group of the metal complex is not disrupted. In the case of hypoxanthine it is also important to emphasize that the absence of D878 is compensated by the mutation of Y885 by an arginine. This allows hypoxanthine to reside in a specific orientation in the active site with the imidazole ring pointing towards the metal complex ready for catalysis.

**Table 6:** Interactions established by MD simulation within the metallic active center in the wild type and variant enzymes.

	E1266-OH	E1266-S1085	Q772-O	D878-Sub	E1266-K889/R885*	R885-Sub
**mAOX3-WT**	a) 1.87±0.16 b) 2.38±0.68	a) 1.79±0.15 a) 3.23±1.73	a) 2.33±0.28 b) 3.12±0.28	a) 5.05±0.48 b) Not available	a) 5.04±1.01 b) 3.29±0.73	Not available
**mAOX3-“active site1”**	a) 5.15±1.25 b) 5.82±0.42	a) 3.76±0.49 b) 1.97±0.32	a) 3.15±0.54 b) 2.22±0.50	a) Not available b) Not available	a) 3.18±0.50 b) 1.81±0.13	a) Not available a) Not available
**mAOX3-“active site1”-K889H**	a) 3.37±1.22 b) 2.17±0.79	a) 1.96±0.29 b) 2.00±0.30	a) 2.12±0.27 b) 2.61±0.42	a) Not available b) Not available	a) 2.17±0.43 b) 1.85±0.16	a) 1.98±0.22 a) 3.70±0.82

*only available in the respective mutated enzymes.

a) substrate is hypoxanthine

b) substrate is benzaldehyde

## Materials and Methods

### Protein Purification and site directed mutagenesis

For site-directed mutagenesis of the presented mAOX3 variants, the expression vector pMMA1 [24] was used as a template. Base pair exchanges were introduced into pMMA1 by PCR mutagenesis. The mAOX3 gene containing base pair exchanges substituting F1014I, F1014V, P1015A and P1015G were cloned into the *Pfl23*II-*Sal*I sites of pMMA1. Exchange of iron-sulfur domains was achieved by PCR-based fusion of the corresponding mAOX1 sequence derived from pSL205 [25] to the appropriate sequence from mAOX3 allowing cloning into the *Nde*I sites of pMMA1. Sequences coding for mAOX3-“active site1” variant (F776K/A807E/D878L/L881S/Y885R/P1015T/Y1019L) and the mAOX3-“gating loop” variant K889H/S1011G/V1012I/G1013S/K1016V/Y1020N) were cloned into the *Pfl23*II*-Spe*I sites of pMMA1. The variant mAOX3-“active site1”-K889H was created by PCR mutagenesis and cloned into the *Pfl23*II*-Spe*I sites of pMMA1. All constructs express the mAOX3 variants as N-terminal fusion proteins with a His_6_-tag. The mAOX3 wild type and variants were expressed and purified as reported previously [24]. The proteins were stored in 100 mM potassium phosphate pH 7.4 at −80°C until usage, without loss of activity. The molybdenum and iron contents were determined by ICP-OES as described previously [24]. The Moco content was quantified after conversion to its fluorescent oxidation product Form A as described in [32].

### Enzyme Assays

Steady state kinetics were performed at 37°C in 50 mM Tris-HCl (pH 8.0) and 1 mM EDTA with variable substrate concentrations (0–250 µM benzaldehyde for mAOX3 wild type, 0–5 mM benzaldehyde for mAOX3 variants, 0–5 mM N1-methylnicotineamide, 0– 400 µM phenanthridine, 0–8 mM hypoxanthine solved in 0.1 M NaOH for mAOX3 wild type, 0–5 mM hypoxanthine solved in 0.1 M NaOH for mAOX3 variants, 0–150 µM hypoxanthine solved in 0.1 M NaOH for bXOR and 0–5 mM allopurinol solved in 0.1 M NaOH) and protein concentrations in a range of 50–200 nM. As electron acceptor, 100 µM 2,6-dichlorophenolindophenol (DCPIP for mAOX3 variants) or molecular oxygen in air-saturated buffer (for phenanthridine) were used in a final reaction volume of 500 µl. Enzyme activity was monitored for 60 seconds at 600 nm for DCPIP and 321 nm for phenanthidine. Specific activity was calculated using the molecular extinction coefficient of 21,400 M^−1^cm^−1^ for mAOX3, 4775 M^−1^cm^−1^ for phenanthridine, and 16,100 M^−1^cm^−1^ for DCPIP [33]. Kinetic parameters were obtained by nonlinear fitting to the Michaelis-Menten model using R, build 2.12.00 [34]. Mean values with standard deviation were obtained from at least 3 independent measurements. k_cat_ was normalized to a 100% molybdenum content.

### EPR-Redox Titration

Anaerobic redox titrations were performed at ambient temperature (24±2°C) under constant mixing under 5% H_2_/95% N_2_ atmosphere (COYlab USA). The potentials were monitored with a platinum combination electrode with Ag/AgCl reference (Pt 5900 A, Schott Instruments, Germany) fitted to an inolab 7310 pH-meter (WTW Germany) calibrated with a saturated quinhydrone solution at pH 7 and 9. Reaction mixture contained 20 µM – 50 µM of protein, 5 µM of mediator and 100 mM potassium phosphate pH 7.4 in a total volume of 2 mL. The mediators used were 2,6-dichloroindophenol (217 mV), phenazinemethosulfate (80 mV), 1,4-naphtochinone (60 mV), toluideneblue (31 mV), methyleneblue (11 mV), indigocarmine (−125 mV), anthrachinone-2-sulfonate (−225 mV), phenosafranine (−252 mV), safranine T (−289 mV) and deiquat (−350 mV). The potential was titrated by stepwise addition of 5 µM–15 µM sodium dithionite (Na_2_S_2_O_4_) dissolved in 100 mM potassium phosphate pH 7.4. After stabilization of the potential (usually after 2 min), 80 µL of sample were withdrawn with a Hamilton syringe, transferred to a 4 mm EPR tube and frozen immediately in liquid nitrogen. 9.5 GHz X-band cw-EPR spectroscopy was carried out on a home-assembled spectrometer consisting of an ER041 MR microwave bridge, a Stanford research lockin amplifier SR810, an AEG magnet with Bruker ER081S power supply and a Bruker SHQ resonator. EPR spectra were recorded at 10 K (for FeSII and FeSI) and 120 K (for Moco) using an Oxford ESR 910 helium flow cryostat controlled by an Oxford ITC502 temperature controller. The microwave frequency was measured with an Agilent 53181A frequency counter. For determination of g-values the magnetic field was calibrated with an external g-standard (LiLiF, g = 2.002293) [35]. Spectra were simulated using the EasySpin Toolbox (Build 4.5.0) and weights fitted to experimental data using Matlab's (R2012) trust-region-reflective non-negative least square algorithm. Weight parameters plotted against the potential were fitted with n = 1 to Nernst equation by non-linear regression using R build 2.12.0 [34].

### UV-VIS-Redox Titrations

Anaerobic redox titrations were performed at 22±1°C (Agilent 89090A #100 peltier) under 5% H_2_/95% N_2_ atmosphere (COYlab, USA). Mixing with 600 rpm was applied for 30 seconds after the addition of dithionite. The potentials were monitored with a platinum combination electrode with Ag/AgCl reference (Pt 5900 A, Schott Instruments, Germany) fitted to an Inolab 7310 pH-meter (WTW Germany) calibrated against saturated quinhydrone solution at pH 7 and 9. Reaction mixtures contained 3 µM–5 µM of protein in 100 mM potassium phosphate pH 7.4 in a total volume of 3 mL. The potential was titrated with stepwise addition of 1 µM–2 µM sodium dithionite (Na_2_S_2_O_4_) solved in 100 mM potassium phosphate pH 7.4. After stabilization of the potential (usually after 2 min), triplicate spectra were recorded on an Agilent 8453 and corrected for the absorbance in the range of 785–800 nm. Absorbance at 444 nm (for FAD) and 550 (for combined [2Fe-2S] centers) plotted against the potential was fitted with n = 2 to Nernst equation by non-linear regression using R build 2.12.0 [34].

### Molecular Docking

All the compounds were studied using the molecular docking software AutoDock [36] and the vsLab plug-in [37]. The structure of the receptor was built from the PDB structure of mouse AOX3 (pdb code 3ZYV at 2.9 Å of resolution). The ligands were built with GaussView [38] and they were protonated at physiological pH. In the docking process we used the Lamarckian genetic algorithm (LGA). The number of generations, energy evaluations, and docking runs were set to 370,000, 1,500,000, and 50, respectively. The types of atomic charges were taken as Kollman-all-atom for the receptor and Gasteiger for the compounds. The final solutions were retrieved from the molecular docking process according to the criteria of interacting energy.

### Molecular Modelling

Since the crystallographic structure of mAOX3 has some gaps in its 3D sequence, the construction of these missing loops was carried out with program Modeller9 version 11 [39]–[42]. The missing parameters of the entire Moco cofactor (molybdenum penta-coordinated to molybdopterin, sulfido, oxo, and hydroxo ligands) were determined. The Moco model structure was optimized using DFT, with the exchange correlation functional B3LYP [43]–[45] and basis set 6-31G+(d) for all atoms except molybdenum, for which the LanL2DZ pseudopotential was employed. A semiflexible model approach was used to calculate the force constants for the bond and angles parameters of the molybdenum metallocenter [46]. Electrostatic charges were determined from a RESP fitting of Merz−Kollman charges [47]. Dihedral force constants involving the Mo were set to zero, while transferable van der Waals atomic parameters were taken from the literature [48]. The parameters for the Fe_2_S_2_ centers were taken from literature too [49].

### Molecular Dynamics Simulation

The structures of the six different complexes (wild type mAOX3:benzaldehyde, wild type mAOX3:hypoxanthine, mAOX3-“active-site1”:benzaldehyde, mAOX3-“active-site1”:hypoxanthine, mAOX3-“active-site1”-K889H:benzaldehyde and mAOX3-“active-site1-K889H”:hypoxanthine) were obtained from the previous docking protocol. For the parameterization of benzaldehyde and hypoxanthine the following protocol was used. The geometries of the molecules were built with GaussView software [38]. The optimized geometries and electronic properties were calculated with Gaussian09 software [50]. The restricted Hartree-Fock (RHF), with the 6-31G(d) basis set was used in all calculations. Atomic charges were recalculated using the RESP algorithm [47]. This methodology was chosen for its consistency with that adopted for the parameterization approach in AMBER 10.0 simulation package [51]. The geometry optimizations and a Molecular Dynamics (MD) simulation on the six complexes were performed with the parameterization adopted in AMBER 10.0, using the parm99SB force field for the protein and cofactors, and the generalized amber force field (GAFF) for the ligands [52].

All hydrogen atoms were added with the Amber software X-Leap [51], taking into account all residues in their physiological protonation state. The only exception was the D878, which was protonated. Twenty counter-ions (Na^+^) were employed to neutralize the high negative charges of the system. The X-Leap program was also used for this purpose. In these simulations, an explicit solvation model with pre-equilibrated TIP3P water molecules was used, filling a truncated octahedral box with a minimum distance of 12 Å between the box faces and any atom of each complex. The average size of each system was 172,000 atoms. The complex geometries were minimized in two stages: first the protein, cofactor and substrate were kept fixed and only the position of the water molecules was minimized. Afterwards the full system was minimized. Subsequently, an MD simulation of 100 ps at constant volume and temperature, and considering periodic boundaries conditions was run, followed by 2 ns of MD simulation with the NPT ensemble, in which Langevin dynamics was used (collision frequency of 1.0 ps^−1^) to control the temperature at 303.15 K [53]. These simulations were carried out using the Sander module. Bond lengths involving hydrogen atoms were constrained using the SHAKE algorithm, and the equations of motion were integrated with a 2 fs time step using the Verlet leapfrog algorithm [54]. The Particle-Mesh Ewald (PME) method [55] was used to include the long-range interactions, and the nonbonded interactions were truncated with a 10 Å cut-off. The MD trajectory was saved every 2 ps and the MD results were analysed with the PTRAJ module of AMBER 10.0 [51].
